# Indiana

**Published:** 1896-06

**Authors:** 


*Indiana.* The Indiana Live-stock Sanitary Commissioners, as a result of heavy losses from hog-cholera, will establish a rigid quarantine this year. Railroad companies will be required to thoroughly disinfect their cars, burn all straw and refuse in stock-yards every seven days, and disinfect the latter at least once a month, under a penalty of $500 in case of violation.

